# Accumulation of Uroporphyrin I in Necrotic Tissues of Squamous Cell Carcinoma after Administration of 5-Aminolevulinic Acid

**DOI:** 10.3390/ijms221810121

**Published:** 2021-09-19

**Authors:** Masatomo Beika, Yoshinori Harada, Takeo Minamikawa, Yoshihisa Yamaoka, Noriaki Koizumi, Yasutoshi Murayama, Hirotaka Konishi, Atsushi Shiozaki, Hitoshi Fujiwara, Eigo Otsuji, Tetsuro Takamatsu, Hideo Tanaka

**Affiliations:** 1 Department of Pathology and Cell Regulation, Graduate School of Medical Science, Kyoto Prefectural University of Medicine, Kyoto 602-8566, Japan; beika@koto.kpu-m.ac.jp (M.B.); minamikawa.takeo@tokushima-u.ac.jp (T.M.); yamaoka@cc.saga-u.ac.jp (Y.Y.); hideotan@koto.kpu-m.ac.jp (H.T.); 2 Division of Digestive Surgery, Department of Surgery, Graduate School of Medical Science, Kyoto Prefectural University of Medicine, Kyoto 602-8566, Japan; nkoizumi@koto.kpu-m.ac.jp (N.K.); murayama@koto.kpu-m.ac.jp (Y.M.); h-koni7@koto.kpu-m.ac.jp (H.K.); shiozaki@koto.kpu-m.ac.jp (A.S.); hfuji@koto.kpu-m.ac.jp (H.F.); otsuji@koto.kpu-m.ac.jp (E.O.); 3 Institute of Post-LED Photonics, Tokushima University, Tokushima 770-8506, Japan; 4 Faculty of Science and Engineering, Saga University, Saga 840-8502, Japan; 5 Department of Medical Photonics, Kyoto Prefectural University of Medicine, Kyoto 602-8566, Japan; ttakam@koto.kpu-m.ac.jp

**Keywords:** 5-aminolevulinic acid, uroporphyrin I, protoporphyrin IX, tumor necrosis, squamous cell carcinoma

## Abstract

5-aminolevulinic acid (5-ALA)-induced protoporphyrin IX (PpIX) fluorescence is widely used for the intraoperative detection of malignant tumors. However, the fluorescence emission profiles of the accompanying necrotic regions of these tumors have yet to be determined. To address this, we performed fluorescence and high-performance liquid chromatography (HPLC) analyses of necrotic tissues of squamous cancer after 5-ALA administration. In resected human lymph nodes of metastatic squamous cell carcinoma, we found a fluorescence peak at approximately 620 nm in necrotic lesions, which was distinct from the PpIX fluorescence peak at 635 nm for viable cancer lesions. Necrotic lesions obtained from a subcutaneous xenograft model of human B88 oral squamous cancer also emitted the characteristic fluorescence peak at 620 nm after light irradiation: the fluorescence intensity ratio (620 nm/635 nm) increased with the energy of the irradiation light. HPLC analysis revealed a high content ratio of uroporphyrin I (UPI)/total porphyrins in the necrotic cores of murine tumors, indicating that UPI is responsible for the 620 nm peak. UPI accumulation in necrotic tissues after 5-ALA administration was possibly due to the failure of the heme biosynthetic pathway. Taken together, fluorescence imaging of UPI after 5-ALA administration may be applicable for the evaluation of tumor necrosis.

## 1. Introduction

Photodynamic diagnosis has been used for tumor detection in the clinical field. Tumor visualization using protoporphyrin IX (PpIX) fluorescence induced by the exogenous administration of 5-aminolevulinic acid (5-ALA) has been applied to brain, bladder, gastric, and esophageal tumors [1,2,3,4,5,6]. PpIX fluorescence at approximately 635 nm allows for the selective visualization of tumor tissues [7,8].

The metabolic pathway of 5-ALA converted to PpIX and heme in cancers has been well studied [2,9]. After the exogenous administration of an excessive amount of 5-ALA, two molecules of 5-ALA are condensed by aminolevulinate dehydratase to form porphobilinogen (PBG) in the cytosol. Four molecules of PBG condense to form hydroxymethylbilane (HMB) by the reaction of porphobilinogen deaminase. Under normal conditions, HMB is converted into uroporphyrinogen III by the action of uroporphyrinogen III synthase (UROS); uroporphyrinogen III is transformed to coproporphyrinogen III. Coproporphyrinogen III consecutively enters the mitochondria, where it is converted to a fluorescent substance of PpIX. PpIX is finally converted to non-fluorescent heme by ferrochelatase [10]. 5-ALA-induced PpIX preferentially accumulates in tumor cells partially due to altered activities of metabolic enzymes in the porphyrin-heme pathway: increased porphobilinogen deaminase activity and suppressed ferrochelatase activity [2,11,12].

Tumor necrosis is a common pathological feature of solid tumors [13,14]. Necrosis often occurs in malignant tumors because of its rapid growth rate and resultant poor blood supply. 5-ALA-induced red fluorescence was shown to be decreased in the necrotic tissues of brain tumors [15]. However, to the best of our knowledge, there has been no report describing fluorescence characteristics and porphyrin metabolism in tumor necrosis.

In this study, we sought to study fluorescence characteristics and porphyrin metabolism in tumor necrosis after the administration of 5-ALA: we investigated excised necrotic tissues of squamous cell carcinoma in a mouse subcutaneous tumor model and human metastatic lymph nodes. The results showed that necrotic tissues have a sharp spectral peak at approximately 620 nm, which differs from that of viable tumor tissues in the mouse model and human metastatic lesions. High-performance liquid chromatography (HPLC) analysis of mouse tissues revealed an alternative metabolite of 5-ALA in necrotic tissues: uroporphyrin I (UPI), with a fluorescence peak at 620 nm, was richly pooled in mouse tumor necrotic lesions. To the best of our knowledge, this study is the first to report on the aberrant accumulation of UPI in tumor necrosis.

## 2. Results

### 2.1. Fluorescence Spectroscopic Analysis of Tumor Necrosis in Clinical Samples

The fluorescence spectra of metastatic lymph nodes of esophageal squamous cell carcinoma were measured ex vivo. Eight patients with esophageal cancer undergoing surgery were enrolled in this study (Table 1). The study population consisted of seven men and one woman with an age range of 56 to 79 years. Seven patients were administered 5-ALA orally 2 h before surgery and one patient 13 h before surgery. The average time between 5-ALA administration and fluorescence imaging was 15.1 ± 5.5 h. No apparent side effects were observed in the study. 

**Figure 1:**
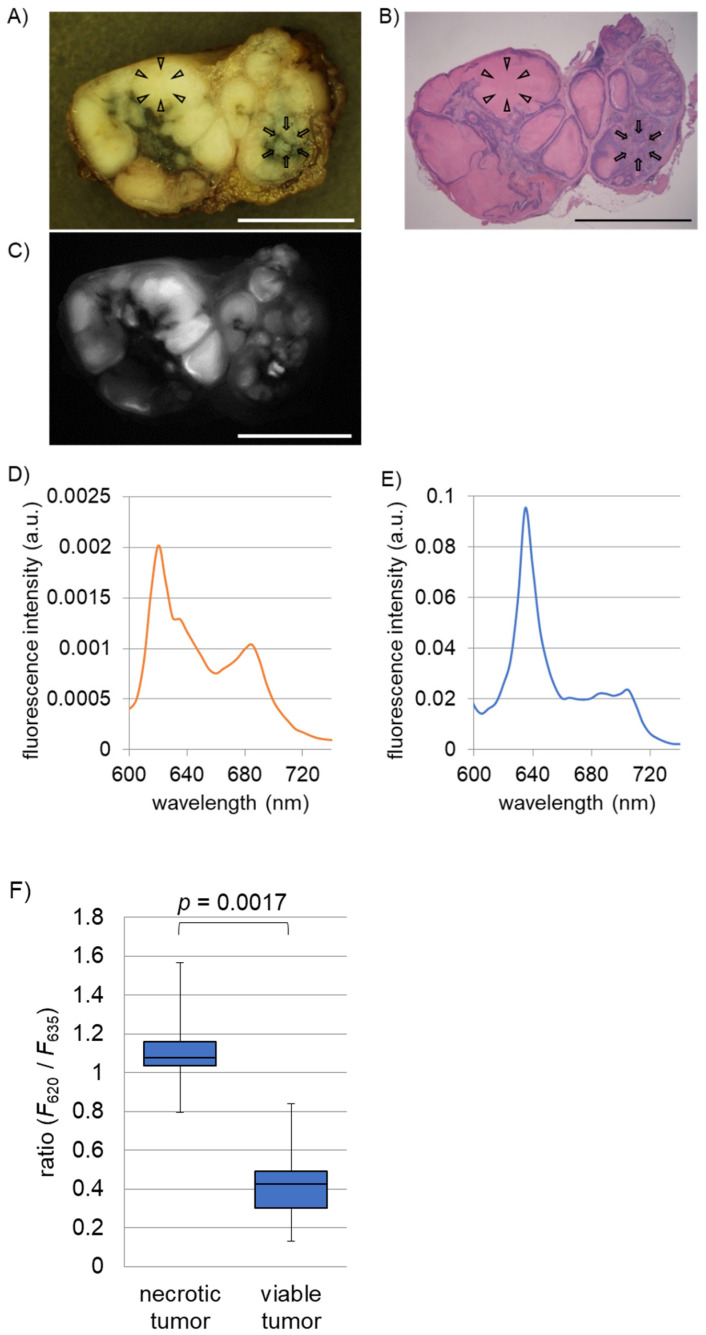
Fluorescence analyses of necrotic and viable tumor tissues of metastatic lymph nodes in esophageal cancer patients. 5-aminolevulinic acid (5-ALA) was orally administered prior to surgery. A total of 16 metastatic lymph nodes excised from eight patients were examined. Representative white-light (**A**), hematoxylin-eosin (HE) staining (**B**), and fluorescence spectroscopic (**C**) images of a metastatic lymph node with tumor necrosis. Note that necrotic (arrowheads) and viable tumor tissues (arrows) are observed. The fluorescence image was acquired at 640 nm. Scale bar, 5 mm. Representative fluorescence spectra of a necrotic tumor lesion (**D**) and viable tumor lesion (**E**). Fluorescence peaks at 620 nm in tumor necrosis and at 635 nm in living tumor are observed. (**F**) Box plot of ratio between fluorescence intensities at 620 nm and 635 nm of tumor necrosis and viable tumors. A significant difference in the ratio between tumor necrosis and viable tumor was indicated (*p* = 0.0017).

A total of 32 metastatic lymph nodes were macroscopically observed ex vivo using white-light imaging. Thereafter, we randomly selected 16 nodes and acquired fluorescence spectra and fluorescence images of necrotic and viable tumor lesions. Of the 16 lymph nodes, 6 were histopathologically diagnosed as metastatic lymph nodes with tumor necrosis and 10 as metastatic lymph nodes without tumor necrosis. We present a representative case of metastatic squamous cell carcinoma with necrosis in an excised human lymph node (Figure 1A–C). Regions indicated by arrowheads and arrows indicate necrotic and viable tumor lesions, respectively. Figure 1C shows a fluorescence image (640 nm ± 10 nm) of the node, showing a bright PpIX fluorescence signal in the viable tumor lesion. Figure 1D,E show representative fluorescence spectra of necrotic and viable tumor lesions. In tumor necrosis, a sharp peak at approximately 620 nm was observed. In contrast, there was a major fluorescence peak at approximately 635 nm in the living tumor lesion. We compared the ratio between fluorescence intensities at 620 nm and 635 nm of necrotic and viable lesions in the 16 lymph nodes, which showed a significantly higher ratio than viable tumor lesions (*p* = 0.0017).

### 2.2. Fluorescence Spectroscopic Evaluation of Tumor Necrosis in a Murine Model

**Figure 2:**
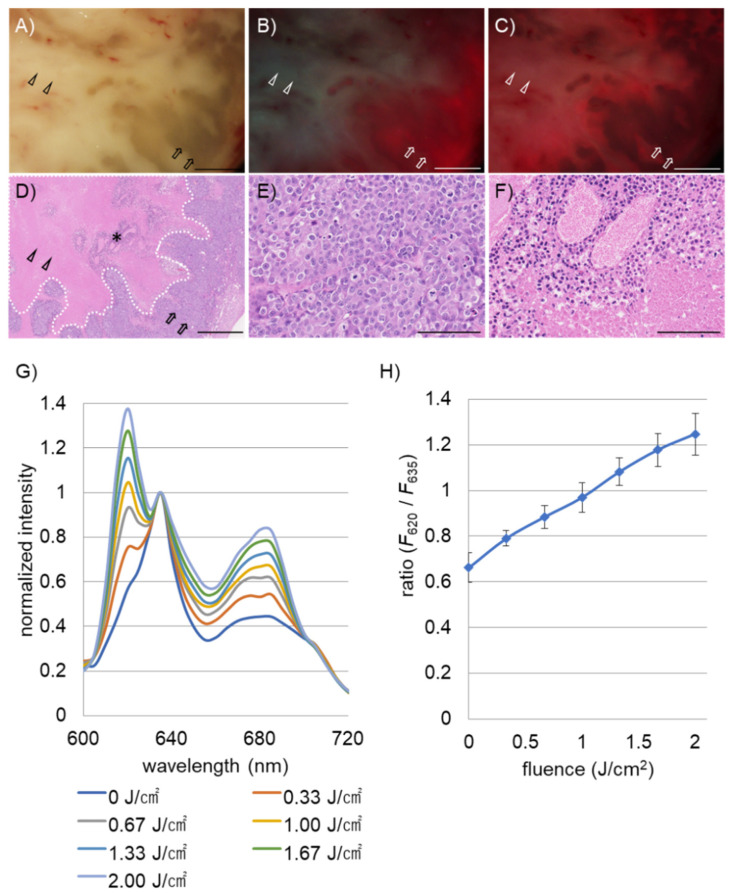
Fluorescence analyses of necrotic and viable tumor tissues resected from B88 squamous cell carcinoma xenografts. Analysis was performed 6 h after the administration of 5-ALA. Representative white-light (**A**), fluorescent (**B**,**C**) (excitation, 405 nm; emission, >430 nm), and HE-stained (**D**–**F**) images of a subcutaneous xenograft. The fluorescence images shown in B and C were acquired before and after irradiation of 405 nm wavelength light (2.00 J/cm^2^), respectively. Arrowheads and arrows denote necrotic and viable tumor lesions, respectively. Note that red fluorescence signal was increased in the necrotic lesion after irradiation (arrowheads in **B**,**C**). Dotted area in D circles necrotic core of the tumor. Degenerated tumor cells around blood vessels were observed in the necrotic core (asterisk in D). Magnified histological images of viable tumor cells, and the degenerated tumor cells in the necrotic core, are shown in E and F, respectively. Scale bar, 1 mm (**A**–**D**) and 100 µm (**E**–**G**). Typical fluorescence spectral change of necrotic lesions after exposures with different fluence of irradiation light. (**H**) Ratio between fluorescence intensities at 620 nm and 635 nm of necrotic lesions as a function of fluence of irradiation light. Each result shown in H represents the mean and standard deviation (error bars) of the experiment performed in triplicate.

We performed fluorescence analyses of necrotic and living tumor tissues resected from B88 squamous cell carcinoma xenografts 6 h after the intraperitoneal administration of 5-ALA. Representative results of the white-light, fluorescence, and hematoxylin-eosin (HE)-stained images of the subcutaneous xenograft are shown (Figure 2A–F). According to histopathological observations (Figure 2D), whitish and brownish regions in the white-light image (Figure 2A) were necrotic lesions (arrowheads) and viable cancer tissues (arrows), respectively. The necrotic core of the tumor is shown circled with a dotted white line (Figure 2D). An enlarged histology showed that viable tumor cells were arranged in sheet-like structures with numerous mitoses outside the necrotic core (Figure 2E). Degenerated cells around blood vessels were observed even in the necrotic core at the microscopic level (asterisk in Figure 2D). Figure 2F shows a magnified view of the area of the asterisk. As shown in Figure 2B, the fluorescence image (emission, >430 nm) before light irradiation revealed that the living tumor tissues generated a strong red fluorescence signal (arrows), while the necrotic tissues emitted a green fluorescence signal (arrowheads). However, the latter produced prominent red fluorescence after irradiation with light (2.00 J/cm^2^) (arrowheads in Figure 2C). Changes in the fluorescence spectra of the necrotic tumor regions upon exposure to irradiation light at 405 nm are shown (Figure 2G). The spectral configuration showed dramatic changes depending on the energy fluence of the irradiating light; the higher the energy, the more recognizable the spectral peak at approximately 620 nm. The fluorescence peak position of necrotic tissues at 620 nm in the murine model was nearly identical to that in the clinically operated samples (Figure 1D and Figure 2G). The relationship between the ratio of fluorescence intensity at 620 nm and 635 nm in necrotic tissues and the fluence of irradiation light is shown in Figure 2H. The ratio increased almost proportionately to the energy fluence of the irradiated light. In addition, we show a representative result of fluorescence spectral change of a viable tumor tissue after exposure to irradiation light (2.00 J/cm^2^) (Figure S1). In the viable tumor, the spectral peak at 620 nm did not appear even after exposure to irradiation light; the height of the spectral peak at 635 nm was markedly decreased owing to the photobleaching effect. Another spectral peak at 675 nm, assigned to photo-protoporphyrin [16], was detected following light irradiation.

### 2.3. HPLC Profiles of Porphyrins in Tissues and Fluorescence Spectra of Pure Chemicals

**Figure 3:**
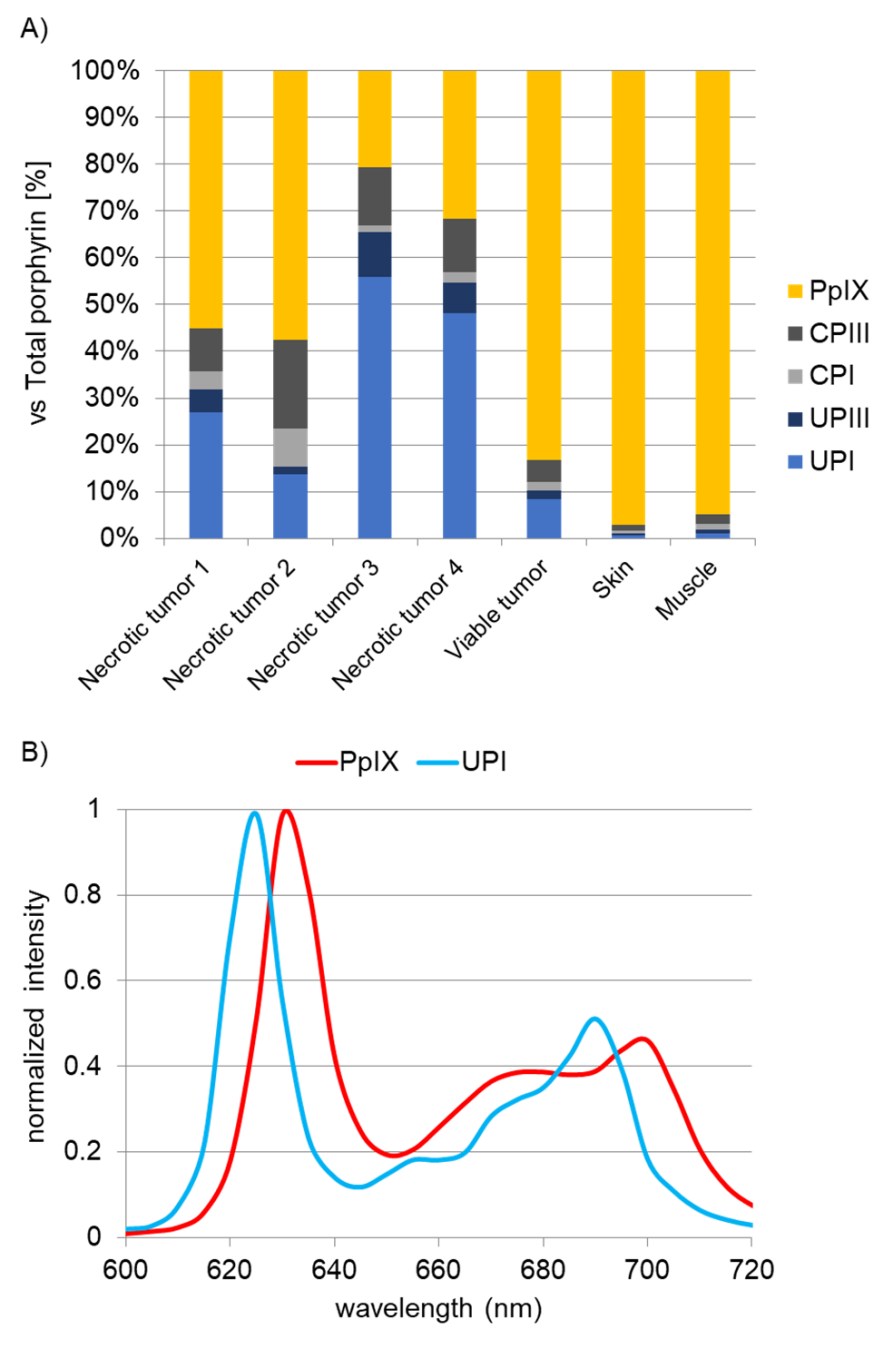
Accumulation of uroporphyrin I (UPI) in tumor necrosis after administration of 5-ALA in a subcutaneous B88 cancer murine model. (**A**) High-performance liquid chromatography (HPLC) analysis. Abundance ratio of each porphyrin to the total amount of porphyrin in necrotic and viable tumors and normal skin and muscle tissues obtained from the xenograft model is shown. Porphyrins including PpIX, CPIII, CPI, UPIII, and UPI were quantified. PpIX, protoporphyrin IX; CPIII, coproporphyrin III; CPI, coproporphyrin I; UPIII, uroporphyrin III; UPI, uroporphyrin I. (**B**) Fluorescence spectra of UPI and PpIX.

Following the fluorescence spectral results, we investigated various porphyrins as a source of the red fluorescence at approximately 620 nm. Then, we performed HPLC analysis of tissues after the administration of 5-ALA with a focus on porphyrins, such as coproporphyrin III (CPIII), coproporphyrin I (CPI), uroporphyrin III (UPIII), and uroporphyrin I (UPI). In the 5-ALA metabolic pathways, uroporphyrinogens I and III, and coproporphyrinogens I and III are non-fluorescent substances, but they form fluorescent porphyrins (UPI, UPIII, CPI, and CPIII) under auto-oxidation and/or photocatalytic reaction, respectively [17]. Necrotic cores and circumjacent viable tumor tissues excised from subcutaneous B88 cancers, normal skin and striated muscle tissues were evaluated, and all samples were excised from the mouse model. Figure 3A shows the results of the abundance ratio of each porphyrin to the total amount of porphyrin in the tissues. UPI was richly detected in necrotic core tissues, whereas PpIX was found predominantly in living tumor, skin, and muscle tissues. In response to the results of the HPLC analysis, we measured the fluorescence spectra of pure chemicals of UPI and PpIX (Figure 3B). Although a main peak in PpIX was observed at approximately 635 nm in PpIX, UPI had a sharp fluorescence peak at approximately 620 nm, and the peak wavelength of UPI was identical to that of the necrotic tissues of the murine model and the clinical sample.

In addition, we sought to obtain UPI and PpIX fluorescence images processed by spectral unmixing to detect necrotic and viable tumors in human lymph nodes (Figure S2). Since a portion of human tissues contain strong autofluorescence-emitting materials of collagen and flavin adenine dinucleotide (FAD), elimination of background autofluorescence signals is required for specifically acquiring 5-ALA-induced fluorescence signals [16,18]. We adopted a spectral unmixing method to resolve crosstalk in multiple fluorescence in tissues, as previously reported [8]. First, we acquired multispectral fluorescence images of the tissues from 480 to 720 nm in 20 nm steps to create spectral unmixed images (Figure S2A). Thereafter, we extracted UPI and PpIX fluorescence signals using spectral unmixing. Spectral unmixing allowed for the visualization of necrotic and viable tumors using UPI and PpIX fluorescence (Figure S2B). We also compared UPI signal intensity in necrotic tumors with that in viable ones in the 16 lymph nodes examined; the former showed significantly higher intensity than the latter (*p* = 0.0048) (Figure S2C).

## 3. Discussion

In this study, we showed that UPI pooled in tumor necrosis of squamous cell carcinoma after 5-ALA administration. According to previous studies, another pathway of 5-ALA metabolism, which is not seen in living cells, has been reported [19,20]. In this pathway, 5-ALA can be non-enzymatically converted to uroporphyrinogen I and UPI. When HMB is condensed by UROS in living cells, it is converted to uroporphyrinogen III. In contrast, if UROS does not work, it spontaneously condenses to produce uroporphyrinogen I. Uroporphyrinogen I is metabolized to UPI by auto-oxidation and/or photocatalysis but not to PpIX. UPI is known to accumulate in the body in congenital erythropoietic porphyria due to enzyme defects in UROS [19].

**Figure 4:**
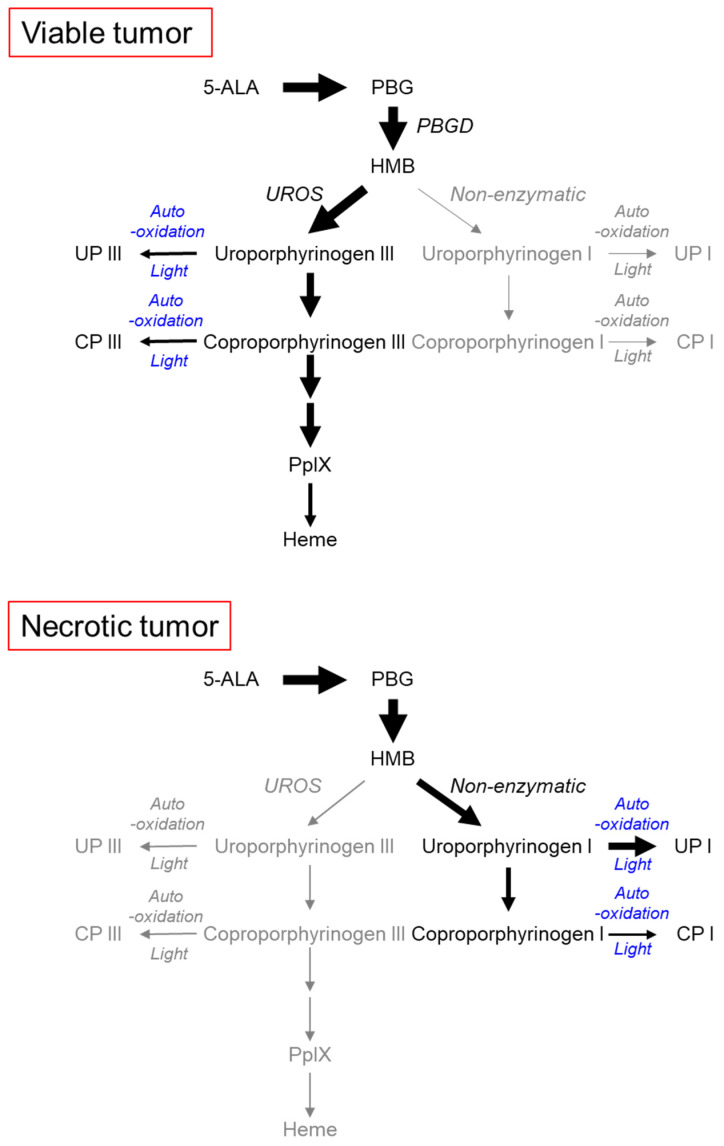
Model of the metabolic pathways of 5-ALA in viable and necrotic tumor tissues. Black and gray drawings in color represent mainstream and non-mainstream pathways, respectively. UPI, UPIII, CPI, and CPIII are generated from respective porphyrinogens by auto-oxidation and/or light-catalyzed reactions. PBG, porphobilinogen; PBGD, porphobilinogen deaminase; HMB, hydroxymethylbilane; UROS, uroporphyrinogen III synthase.

Unexpectedly, we found red fluorescence at 620 nm in tumor necrosis of the metastatic lymph nodes and the subcutaneous tumor model (Figure 1 and Figure 2). In HPLC analysis, we analyzed 5-ALA metabolites in the living and necrotic regions of mice after light irradiation. HPLC analysis revealed rich PpIX and UPI in living tumor tissues and necrotic tissues, respectively (Figure 3A). In living tumor tissues, a large amount of PpIX was found because the heme biosynthetic pathway of the uroporphyrinogen III/coproporphyrinogen III axis is active (upper panel of Figure 4) [2,9]. In contrast, UPI was abundantly produced in the necrotic tissues. In tumor necrosis, the spontaneous non-enzymatic pathway from HMB to uroporphyrinogen I seemed to be dominant compared to the pathway from HMB to uroporphyrinogen III, resulting in the production of uroporphyrinogen I, a precursor of UPI (lower panel of Figure 4) [17,19,20]: UPI was readily generated under auto-oxidation and/or light-catalyzed reaction [17,19].

Although UPI was rich in tumor necrosis, UPIII, CPIII, and PpIX were also present, even in the necrotic core tissues (Figure 3A). This may be because we identified necrotic and living tumor masses for HPLC analysis using a macroscope. We made a pathological diagnosis based on 4 µm-thin sections with HE staining to identify tumor necrosis, as shown in Figure 2D–F, but HPLC analysis of UPI, UPIII, CPI, CPIII, and PpIX was not performed at the microscopic level. For HPLC analysis, we needed a minimum of 0.60 g of necrotic tissue blocks excised from at least five mice. There is a possibility of contamination of living cells, even in the tissue blocks macroscopically identified as necrosis. As shown in Figure 2F, there seems to be a relatively small amount of degenerated cells, even in the necrotic core of the tumors. This may be why UPIII, CPIII, and PpIX fluorescence, which should be observed in living cells, were present even in the necrotic core tissues.

In the analysis of human metastatic lymph nodes, we found a spectral peak at approximately 620 nm without irradiation of light for the catalyzed reaction (Figure 1D). In the experiments with the murine model, we did not clearly detect the 620 nm peak before exposure to irradiation light (Figure 2G). This could be explained by the following reasons: (1) UPI can be produced from uroporphyrinogen I under auto-oxidation reactions without the aid of photocatalysis [19,20]. Moreover, the long period of time between 5-ALA administration and fluorescence imaging due to long operative durations might have an impact on the formation of UPI: the average time between 5-ALA administration and fluorescence imaging was 15.1 h. In contrast, fluorescence analyses in mice were performed 6 h after 5-ALA administration; (2) Lighted environments in operating theaters for esophageal cancer patients might enhance the formation of UPI from uroporphyrinogen I, although we could not clearly demonstrate the relationship between the peak strength at 620 nm and irradiated light fluence during operations. Therefore, further studies are required.

Regarding the translational aspects of this study, the accurate intraoperative measurement of tumor necrosis induced by chemotherapy may lead to improved esophageal cancer treatment outcomes [21]. The effects of neoadjuvant chemotherapy for esophageal cancer differ greatly between primary tumors and metastatic lymph nodes. Metastatic lymph nodes reflect the spread of tumors throughout the body, and the lymph node response to neoadjuvant chemotherapy predicts long-term survival more precisely than the primary tumor response in patients with metastatic esophageal cancer. To identify necrotic and viable tumors, fluorescence imaging of UPI and PpIX has the potential to be applied in clinical settings. UPI and PpIX fluorescence processed by spectral unmixing distinguished necrotic lesions from viable ones (Figure S2), and we previously reported that the spectral unmixing method is useful for resolving crosstalk in multispectral fluorescence images of 5-ALA-administered tissues [8].

Our study has some limitations. First, we were unable to estimate the enzymatic activities of each heme biosynthetic pathway, for example, UROS. Further evaluation will be required in the near future. Second, we did not perform fluorescence analysis after the photocatalytic reaction or HPLC experiments in the human lymph nodes. In this study, there was insufficient tissue available for additional experiments to ensure accurate pathological examination. Lastly, we studied only tumor necrosis in the resected tissues. Intraoperative fluorescence analysis is needed as a future endeavor.

In conclusion, we demonstrated that UPI accumulated in tumor necrotic tissues after 5-ALA administration. This indicates that non-fluorescent uroporphyrinogen I, a precursor of fluorescent UPI, was generated in tumor necrosis. Our study suggests that fluorescence imaging of UPI can be used to evaluate the coagulative necrosis of tumor tissues after 5-ALA administration. We plan to examine other types of malignant tumors, including gliomas and urothelial carcinomas. These approaches would highlight the potential of 5-ALA for intraoperative diagnosis of tumors.

## 4. Materials and Methods

### 4.1. Chemical Agents

PpIX (Alexis Biochemicals, San Diego, CA, USA) and UPI dihydrochloride (Wako Pure Chemical Industries, Ltd., Osaka, Japan) were dissolved in dimethyl sulfoxide at a concentration of 1.0 mM and kept frozen in a light-proof bottle until just before use. 5-ALA for use as patient medication was purchased from Cosmo Bio Co., Ltd. (Tokyo, Japan).

### 4.2. Clinical Study

A clinical trial intended for patients with esophageal cancer was performed in accordance with the ethical code of our institution from March 2011 to January 2013. The study was approved by the Ethics Committee of Kyoto Prefectural University of Medicine (RBMR-C-671, published on 19 April 2010). Exclusion criteria were passage obstruction due to pyloric stenosis, history of porphyria, history of allergy, renal insufficiency, and hepatic insufficiency. 5-ALA at 15–20 mg/kg dissolved in 20 mL of 50% glucose was administered to the patients orally prior to surgery. Patients were protected from direct sunlight for 24 h after 5-ALA application to avoid photosensitization. Periesophageal lymph nodes were removed and transported to the laboratory to avoid light exposure and then analyzed. Lymph nodes were cut in half using a fine razor. Fluorescence spectral and imaging analyses of cut slices were performed.

### 4.3. Cell Culture and Tumor-Bearing Mouse Model

The human oral squamous cell carcinoma cell line, B88 cells [22,23,24], was kindly provided by Prof. Daisuke Uchida (Department of Oral Surgery, Subdivision of Molecular Oral Medicine, Division of Integrated Sciences of Translational Research, Institute of Health Biosciences, The University of Tokushima Graduate School). The cells were cultured in Dulbecco’s modified Eagle’s medium (Wako Pure Chemical Industries, Ltd., Osaka, Japan) supplemented with 10% fetal bovine serum and 1% penicillin-streptomycin in a humidified 5% CO_2_ incubator. A subcutaneous tumor model of squamous cell carcinoma was prepared using B88 cells. Briefly, female BALB/c nude mice (5–8 weeks old) were injected subcutaneously into the cells (1 × 10^7^ cells). After injection, the mice were fed regularly. We limited the maximum tumor burden to 17 mm at its largest dimension and maximum tumor volume to less than 10% body weight according to the guidelines of the United Kingdom Co-ordinating Committee on Cancer Research (UKCCCR). The animal experiments were approved by the Ethical Committee of Kyoto Prefectural University of Medicine (M24-051, published on 1 April 2012).

### 4.4. 5-ALA Treatment of Mice

The mice were intraperitoneally administered a dose at 250 mg/kg body weight of 5-ALA hydrochloride (Wako Pure Chemical Industries, Ltd., Osaka, Japan) 6–8 weeks after tumor inoculation. Six hours after administration, the excised subcutaneous tumors on the backs of the mice were cut in half. The cut surfaces were examined using fluorescence microscopy and spectroscopy. For HPLC analysis, specimens were collected after necrotic cores and peripheral viable tumors were macroscopically judged by the appearance of the tumors cut in half.

### 4.5. Fluorescence and Histological Analysis

Fluorescence spectra were acquired using a fluorescence analytical system composed of a microscope (SZX12; Olympus, Tokyo, Japan), an intensified multichannel spectrophotometer (MCPD-7000; Otsuka Electronics, Osaka, Japan), and a mercury lamp (ULH100HG; Olympus, Tokyo, Japan), as described in previous studies [16,25]. A band-pass filter of D405/20× (excitation, 405 nm ± 10 nm) (Chroma Technology Corp., Rockingham, VT, USA) was used for excitation and irradiation. Excitation and irradiation lights were used to acquire fluorescence images and induce photocatalytic reactions, respectively. The measurement of each spectrum was repeated five times, and the averaged spectrum was acquired. Fluorescence images were acquired using a color CCD camera (DP71; Olympus, Tokyo, Japan) under 405 nm excitation light through a long-pass filter (>430 nm) (HQ430LP; Chroma Technology Corp., Bellows Falls, VT, USA). White-light images were recorded by microscopes (SZX12 for murine samples (Olympus, Tokyo, Japan) and MVX10 for human samples (Olympus, Tokyo, Japan)) equipped with halogen lamps (LG-PS2; Olympus, Tokyo, Japan). The fluorescence images (640 nm ± 10 nm) were taken using a system consisting of a fluorescence microscope (MVX10; Olympus, Tokyo, Japan) equipped with a monochrome charge-coupled device camera (ORCA-ER; Hamamatsu Photonics, Hamamatsu, Japan), a tunable filter (Varispec; CRi, Woburn, MA, USA), and a mercury lamp, as described previously [8]. A fluorescence filter cube composed of an excitation bandpass filter (BP400-410; Olympus, Tokyo, Japan), a barrier filter with a cutoff of 455 nm (BA455; Olympus, Tokyo, Japan), and a dichroic mirror (DM455; Olympus, Tokyo, Japan) were attached to the microscope.

After fluorescence spectral and image acquisition, all observed samples were fixed with 10% formalin. Histological sections substantially equivalent to the observed surface were made, and histopathological diagnosis was performed based on HE staining by experienced pathologists with no knowledge of the results of fluorescence observation.

### 4.6. HPLC Analysis

The accumulation of 5-ALA-induced porphyrins in necrotic and viable tumor tissues and muscular and skin tissues of tumor-bearing mice was examined using HPLC analyses. We applied a previously described method [26], with some modifications. Each excised frozen tissue (0.60‒1.63 g in total) was homogenized in four times its volume of phosphate-buffered saline. Each specimen, that is, necrotic tumor 1, necrotic tumor 2, necrotic tumor 3, necrotic tumor 4, viable tumor, skin, and muscle, as shown in Figure 3A, consisted of tissues excised from at least five mice. Twenty tumors excised from 20 mice were analyzed. Then, 0.10 mL of the homogenate, 0.01 mL of 50% acetic acid (*v*/*v*), and 0.30 mL of N, N-dimethylformamide-2-propanol (DMF-IPA) solution (100/1, *v*/*v*) were mixed. After centrifugation, the supernatants were collected. Then, 0.15 mL of DMF-IPA solution was added to the precipitate again, and the same operation was repeated. A mixture of the two obtained supernatants was used as the porphyrin extraction solution. 

HPLC analysis of PpIX was performed under the following conditions: each prepared porphyrin extraction solution was injected into an Alliance HPLC system (Waters Corporation, Milford, MA, USA) equipped with a CAPCELL PAK UG120 C18 column (5 μm, φ4.6 × 150 mm; Shiseido, Tokyo, Japan). The elution solution was a mixture of acetonitrile and 10 mM tributylamine (pH 7.5) (70:30, *v*/*v*). The fluorescence signal (excitation, 400 nm; emission, 630 nm) was detected.

For the HPLC analysis of UPI, UPIII, CPI, and CPIII, we employed the following conditions: A mixture of 0.10 mL of the above mentioned porphyrin extraction solution and 0.05 mL of acetic acid was injected to the Alliance HPLC system equipped with a Symmetry C18 column (5 μm, φ3.9 × 150 mm; Waters Corporation). The elution solutions were solvent A (1 M ammonium acetate, including 10.5% acetonitrile, pH 5.15) and solvent B (50 mM ammonium acetate, including 80% acetonitrile, pH 5.15). The elution was first performed with solvent A for 10 min, followed by a linear gradient of solvent B (20–27.2%) for 14.5 min, followed by elution with solvent B for 10 min. The fluorescence signals (excitation, 404 nm; emission, 620 nm) were analyzed. The porphyrin concentrations in the specimens were evaluated using calibration curves obtained using standard porphyrins.

### 4.7. Statistical Analysis

Quantitative data were expressed as box plots. Statistical significance was evaluated using the Mann-Whitney U test.

## Figures and Tables

**Table 1:** Clinicopathological features of enrolled patients with esophageal cancer.

Case Number	Age (Years)	Sex	Histology	Tumor Location	Tumor Depth (T)	Nodal Status (N)	Stage	Pre-Operative Chemotherapy
1	74	Male	SCC	C15.4	T3	N1	III	5-FU/CDDP
2	57	Male	SCC	C15.0	T3	N1	III	5-FU/CDDP
3	76	Male	SCC	C15.3	T3	N3	IVA	S-1
4	79	Female	SCC	C15.0	T3	N1	III	(-)
5	64	Male	SCC	C15.4	T3	N1	III	5-FU/CDDP
6	67	Male	SCC	C15.5	T3	N3	IVA	5-FU/CDDP
7	72	Male	SCC	C15.4	T2	N3	IVA	5-FU/CDDP
8	56	Male	SCC	C15.5	T3	N1	III	5-FU/CDDP

SCC, squamous cell carcinoma; 5-FU, 5-fluorouracil; CDDP, cisplatin; S-1 (a fourth-generation oral fluoropyrimidine), Tegafur-Gimeracil-Oteracil potassium capsule; C15.0, cervical esophagus; C15.3, upper thoracic portion; C15.4, mid-thoracic portion; C15.5, lower thoracic portion; classified according to the 6th edition of the UICC TNM classification for esophageal cancer.

## Supplementary Materials

The following are available online at https://www.mdpi.com/article/10.3390/ijms221810121/s1.
