# Supplementary material for: Accumulation of Uroporphyrin I in Necrotic Tissues of Squamous Cell Carcinoma after Administration of 5-Aminolevulinic Acid
Source: Int J Mol Sci. 2021 Sep 19;22(18):10121. doi: 10.3390/ijms221810121 (PMC8471361; doi:10.3390/ijms221810121)
Supplement: Supplementary file 1 [file ijms-22-10121-s001.zip › ijms-1345055.pdf]

Beika M, *et al.*

Supplementary data

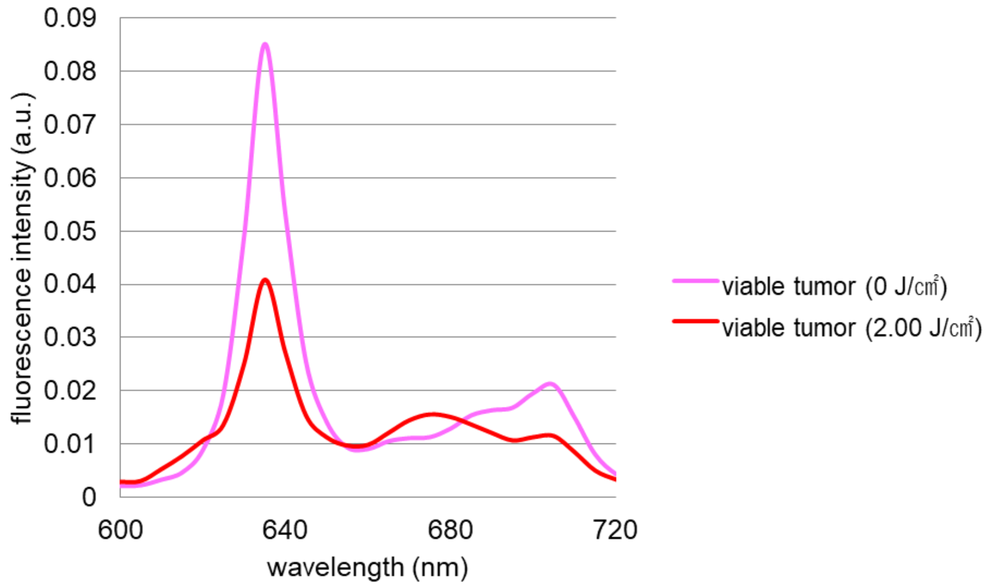

Figure S1. Fluorescence spectral change of viable tumor tissues after irradiation (2.00 J/cm^2). Representative spectra without normalization are shown. The tumor tissue was excised from a B88 squamous cell carcinoma xenograft. Analysis on half-cut tumor was performed 6 h after the administration of 5-aminolevulinic acid (5-ALA).

A)

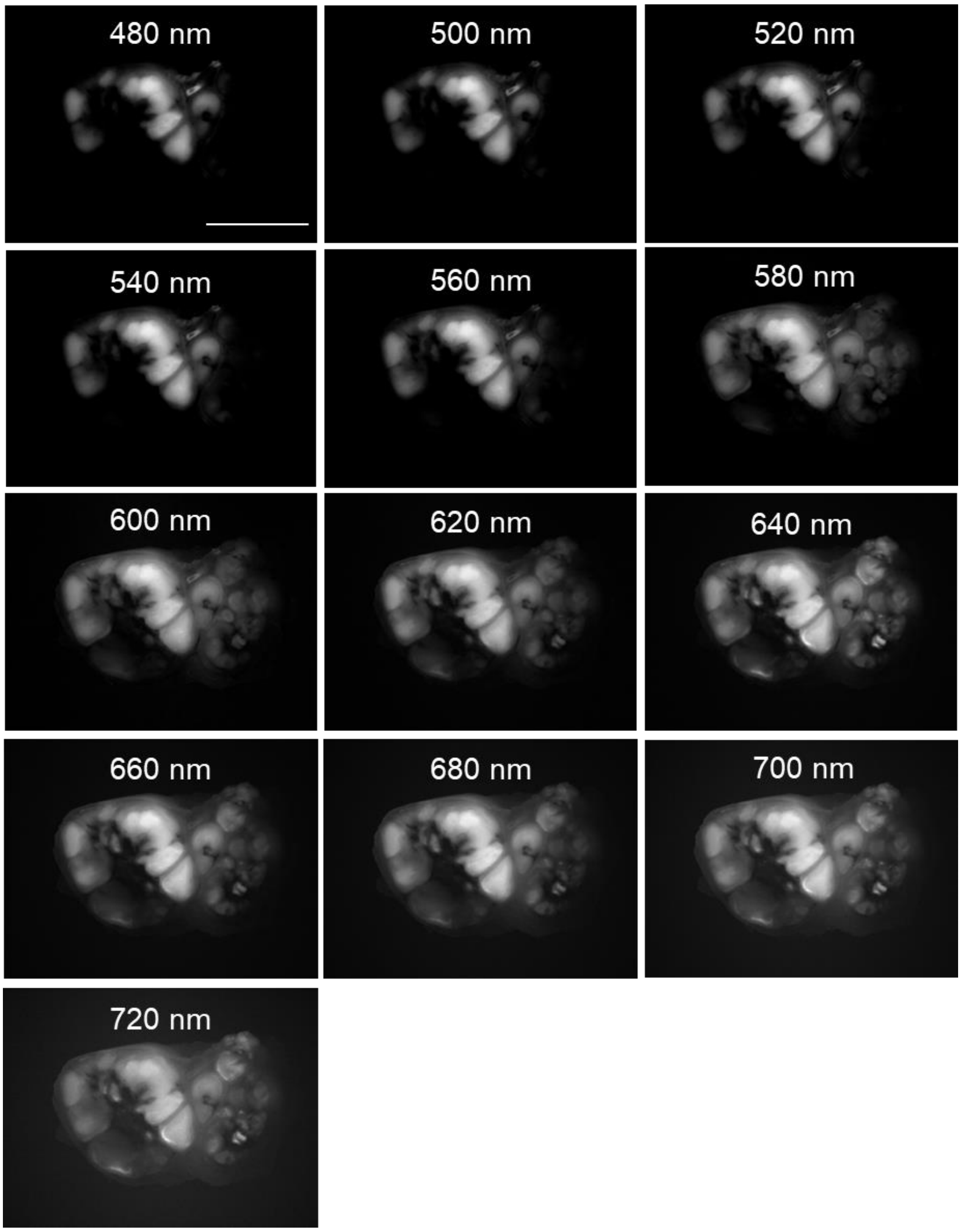
480 nm
500 nm
520 nm
540 nm
560 nm
580 nm
600 nm
620 nm
640 nm
660 nm
680 nm
700 nm
720 nm

B)

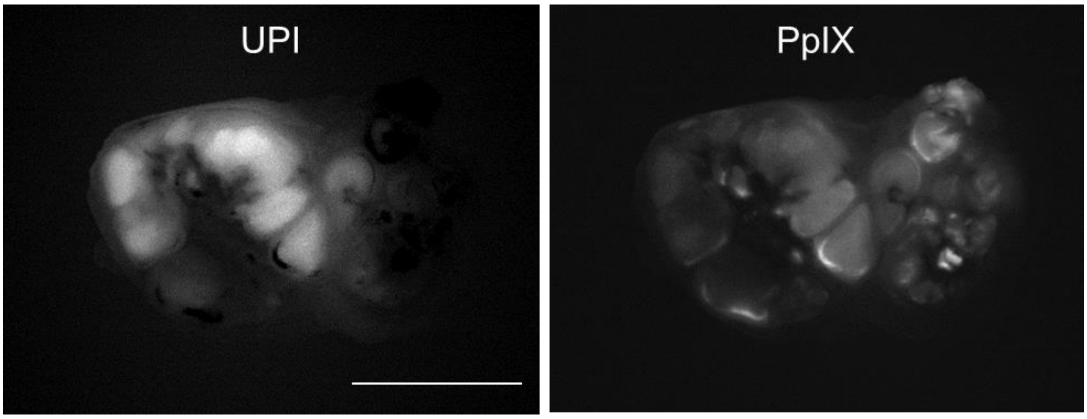

C)

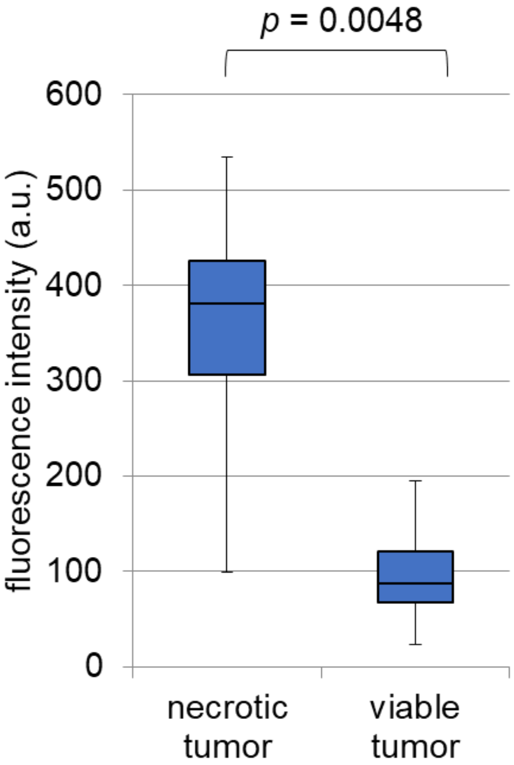

Figure S2. Fluorescence analyses of necrotic and viable tumors of lymph nodes in esophageal cancer patients by using spectral unmixing. 5-ALA was orally administered prior to surgery. A total of 16 metastatic lymph nodes excised from eight patients were examined. (A) Multispectral fluorescence spectroscopic images of a metastatic lymph

node with tumor necrosis. The images were acquired from 480 to 720 nm in 20-nm steps for spectral unmixing. The image acquired at 640 nm are a duplicate of Figure 1C. See Figure 1A and 1B for location of the necrotic and viable tumors. Scale bar, 5 mm. (B) Spectral unmixed fluorescence images of uroporphyrin I (UPI) (left panel) and protoporphyrin IX (PpIX) (right panel). Scale bar, 5 mm. (C) Box plot of UPI fluorescence intensities of necrotic and viable tumors. The signal intensities for the unmixed images of UPI were analyzed in the 16 lymph nodes. A significant difference in the intensity between tumor necrosis and viable tumors was indicated ($p = 0.0048$).

Materials and Methods

Fluorescence spectral analysis

Fluorescence spectra of the B88 tumor were acquired using the system described in the manuscript.

Spectral unmixing

Spectral unmixing analysis of 5-ALA induced fluorescence in tissues has been described elsewhere [1]. Briefly, the method assumes that the signal intensity of each pixel in an obtained image can be expressed by a linear combination of fluorescence spectra from several substances with known spectra [2]. Multispectral fluorescence images for spectral unmixing were acquired using the system described in the manuscript. Multispectral fluorescence images were acquired from 480 nm to 720 nm in 20-nm steps. 1.0 mM PpIX (Alexis Biochemicals, San Diego, CA, USA), 1.0 mM UPI dihydrochloride (Wako Pure Chemical Industries, Ltd., Osaka, Japan), 90 mM flavin adenine dinucleotide (FAD) (Wako Pure Chemical Industries, Ltd., Osaka, Japan), and collagen powder from

bovine Achilles tendon, type I (Sigma-Aldrich, St. Louis, MO, USA) were used as the user-defined reference spectra for spectral unmixing. The intensity values of PpIX, UPI, FAD, and collagen on each pixel in tissues were estimated by plotting the score of the pure chemical of PpIX, UPI, FAD, and collagen according to the calculated concentration matrix. Of the four fluorescent substances, a two-dimensional distribution of intensity values of PpIX and UPI was created.

Statistical analysis

The fluorescence intensity of the UPI was evaluated using the maximum value of the unmixed images. Quantitative data were expressed as box plots. Statistical significance was evaluated using Mann-Whitney U test.

References

1. Harada, K.; Harada, Y.; Beika, M.; Koizumi, N.; Inoue, K.; Murayama, Y.; Kuriu, Y.; Nakanishi, M.; Minamikawa, T.; Yamaoka, Y.; Dai, P.; Yanagisawa, A.; Otsuji, E.; Takamatsu, T., Detection of lymph node metastases in human colorectal cancer by using 5-aminolevulinic acid-induced protoporphyrin IX fluorescence with spectral unmixing. *Int J Mol Sci* **2013,** 14, 23140-23152.
2. Garini, Y.; Young, I. T.; McNamara, G., Spectral imaging: principles and applications. *Cytometry A* **2006,** 69, 735-747.
